# Medical Opinion and Movement

**Published:** 1909-05-15

**Authors:** 


### Medical Opinion and Movement.

The Treatment of Nocturnal Enuresis by Thyroid Extract has been tested by Dr. Leonard Williams, who publishes a series of 25 cases in the *Lancet*. He was led to try this remedy by the entire failure of the usual measures in the case of a public schoolboy who would have been obliged to leave his school if the trouble had continued. On this patient circumcision had been performed in infancy, and a small collection of adenoids was removed by a specialist: so neither phimosis nor nasal obstruction could be held accountable for the enuresis. In fact, after the operation for adenoids the nocturnal discharge of urine became very much more frequent. This suggested to Dr. Williams that the removal of a mass of adenoid tissue had deprived the lad of something which counteracted the abnormal condition of the nervous system causing sphincter relaxation. So on the possibility that the most similar tissue known, that of the thyroid gland, might restore the lost balance, he ordered thyroid gland extract, with results apparently magical. The boy, it is to be added, showed no signs either of hyperthyroidism or cretinism, though he was somewhat below the average height and weight for his age. Having achieved such striking success, Dr. Williams proceeded to investigate an extended series of hospital cases, with results equally satisfactory. Only one of his 25 patients obtained no benefit from the treatment, though not all were cured with the suddenness and completeness of the public-school boy. On searching the literature very carefully for previous investigations on this subject, Dr. Williams came across only one allusion to the value of thyroid medication of enuresis, that of a Belgian physician. Considering the refractory nature of this complaint to the hitherto accepted lines of treatment, the series now reported is one in which all practitioners must be interested, and further results along these lines will be eagerly awaited.

---

Some interesting remarks on Scurvy are contained in a paper recently published by Dr. G. A. Turner, medical officer to the Witwatersrand Native Labour Association. The subject under consideration is the diet of South African native races in their own kraals, and after noting the extraordinary powers of endurance of many tribes on apparently most inadequate diet, the author remarks that if after such privation they are given severe muscular labour on a full and highly nutritious dietary, they seem to be especially prone to contract scurvy. This peculiar sequence has been observed amongst the Maherero, who flocked to the Rand after the uprising against the German authorities in Damaraland; in natives from Bechuanaland at Kimberley after the campaign in that country; after the Matabele rebellions, and so on. An interesting point is that when scurvy thus occurs, lime juice seems to have little or no curative effect upon it. It is pointed out that the negro in these circumstances has been deprived especially of proteids, whereas the sailor on board ship may have had sufficient or even too much proteid in his food, but not enough vegetable salts. Bearing this in mind, it is interesting to recall that on Capt. Robert Scott's Antarctic expedition five or six years ago such cases of mild scurvy as occurred reacted much more satisfactorily to the provision of fresh seal meat than to any system of lime or other vegetable therapy. Dr. Turner, curiously enough, has himself noticed that the best treatment of the scorbutic natives he has had under care is to add raw meat to the soup which they are given. The question of the exact causation and treatment of scurvy is one in which little progress has been made of late years, largely owing to the success with which prophylaxis has prevented this once common disorder.

---

"The Cure of Typhoid" is the somewhat startling title of a paper by Mr. J. Maberly in the *Transvaal Medical Journal*. After pointing out that the present expectant treatment of typhoid fever is analogous to that of acute rheumatism before the introduction of salicylate compounds, and that there is, therefore, no *a priori* objection to the empirical use of any new drug, he proceeds to give his experience with tincture of monsonia biflora, and with an active principle which has been isolated from it and named entericin. It is claimed for the tincture that it is particularly valuable for hæmorrhage in typhoid fever, and for any case in which distension or profuse diarrhœa suggest the possible imminence of that complication. The action of this drug in doses up to three drachms two-hourly is regarded both by the author and by Dr. Darley-Hartley as practically specific. The great objection which is found to militate against the use of monsonia as a routine part of the treatment of typhoid fever patients is its markedly constipating effect. To get over this objection the aid of analytical chemists was invoked, and a body has been isolated of stable but indefinite composition which is believed to contain the active principle without any constipating action. As a result of a somewhat limited experience since this body was isolated, Mr. Maberly has strong hopes that he has solved the problem of the curative treatment of typhoid; for he suggests tentatively that entericin has an antidotal action on the typhoid toxin itself, as well as some specific effect upon the actual ulcers. It is to be noted that the four published cases were all of fairly severe type, that the fever subsided rapidly and permanently in each case, and that a much more liberal dietary was ordered than is usually allowed in this country.

---

An interesting discussion took place at a recent meeting of the Medical Society of Paris on the Pathology and Treatment of Neurasthenia. Dr. Godleski sought to define clearly and distinguish neurasthenia from other neuropathic conditions with which it is often confounded. True neurasthenia, he maintained, is always due to an intoxication produced either by physical, intellectual, or especially moral fatigue, or by some previous malady of an infective nature. This intoxication leads to nutritive disturbances of the cells of the body, and especially of the nervous system. Hence the rational treatment of neurasthenia should be directed towards a disintoxication and reconstitution of the cells. He did not deny the mental or psychical factor involved in the disease, but the weakening of volition is only a result of the physical morbid condition. Psycho-therapy, persuasion, re-education of the will are of value, but they can only be regarded as complementary, and do not form the basis of rational treatment.

---

An instance of Gastric Ulcer caused by Needles in the stomach is reported by Fuld and Katzenstein. A young girl in good health had swallowed by mistake some needles she had in her mouth. Two months later she consulted Dr. Fuld on account of gastric pain, and he detected a circumscribed sensitive area in the pyloric region on deep palpation. Radiographic examination revealed nothing, but as the pain persisted in spite of treatment, operation was determined upon. Katzenstein discovered on opening the stomach two needles placed at an angle of forty-five degrees to each other, about four centimetres from the pylorus. There was some thickening around, and in the centre a granulating ulcer. A third needle was also found embedded in the muscular coat. The only indication of its presence was a longitudinal cicatrix in the mucous membrane. Having removed the needles Katzenstein scraped the granulating surface and covered it with normal mucous membrane. The patient recovered completely, and had no recurrence of pain.

---

Dr. G. Auzilotti, of Pisa, advocates the intramuscular injections of Gelatin combined with Chloride of Calcium, as a means of assisting ossification in cases of Fracture with delayed consolidation, chronic osteo-myelitis and pseudo-arthrosis. He uses a 2.5 per cent. sterilised solution of gelatin with .75 per cent. sodium chloride, .5 per cent. phenol, and .5 to 1 per cent. of calcium chloride. He injects 10 c.c. of this solution every day or every other day into the muscles of the buttock. He has carried out the treatment in a large number of cases, and is convinced of its efficacy in favouring the processes of osseous repair. The injections are quite harmless, and cause no local reaction. The method was first used by Dr. V. Colla in a case of general osteo-malacia with multiple fractures. The condition appeared to be ameliorated by the treatment, and there was reunion of the fractures. Gelatin is known to cause an increase in the coagulability of the blood, but whether there is any connection between this phenomenon and the formation of osseous tissue, or what the underlying physiological processes are by which the gelatin is able to influence the process of bone repair have not hitherto been explained.

---

In *Le Progrès Médical*, Dr. G. Milian discusses the value of the Corneal Reflex in cases of coma of doubtful origin. In such cases the question often presents itself whether the coma is due to an intoxication, a cerebral lesion, or whether it is a post-epileptic coma, or a coma of hysterical origin. The condition of the corneal reflex will generally give a decisive answer. In cases of extreme intoxication it may be very feeble or absent, but it will be the same on both sides, although the author makes the interesting observation that in cases of narcosis from chloroform, careful observation will reveal the fact that the corneal reflex will disappear from one side first. In cases of hemiplegia, however, the corneal reflex is abolished only on the paralysed side, and similarly in Jacksonian epilepsy there may be absence of the corneal reflex only on the side on which the convulsions appear, to be followed by abolition of both reflexes when the convulsions become generalised. This may afford an important diagnostic sign in the differentiation of Jacksonian epilepsy from hysterical convulsions or true epilepsy. The author cites an interesting case of a woman addicted to morphia injections in large doses, who became suddenly comatose, and the diagnosis of morphia intoxication was made. The author, however, observed a marked immobility and inertness of the limbs of the left side of the body compared with the right. Babinski's sign was indecisive, and the knee-jerks were present on both sides, but there was complete absence of the corneal reflex on the left side, and this enabled him to make a definite diagnosis of hemiplegia.

---

The Appendices Epiploicæ play a by no means unimportant rôle in abdominal pathology. They may produce internal strangulation, they may themselves become strangled in herniæ and they may become detached as a result of previous torsion, and form foreign bodies in the peritoneal cavity. Torsion of the appendices is the most common form of accident observed, but this has hitherto always been of a chronic nature. Dr. H. Zöppritz, of Kiel, however, reports a case of acute primary torsion of an appendix epiploica which was verified and relieved by operation. The patient, a young man, had for some time suffered from abdominal cramps more or less intense on certain movements of the body. One night he was seized suddenly with extremely acute pain just above the umbilicus. The pain persisted during the following day, and was accompanied by some prostration. There was some resistance of the abdominal wall over the painful region. The temperature was slightly raised, pulse 96, and good, and no nausea or vomiting. Operation was resorted to, and the usual incision for appendicectomy was performed, but the appendix was found to be quite healthy, but on carrying the incision higher up, a small bluish-red mass was discovered, surrounding adhesions were detached, and a little tumour of the size of a prune presented itself as an appendix epiploica of the transverse colon, twisted on its pedicle to an angle of 180 degrees. This was ligatured and resected and the patient was cured.

---

Kuttner has recently published an account of a series of 21 Thoracic Operations performed by himself in a Chamber in which the Atmospheric Pressure can be Raised or Lowered at will. Under diminished pressure he operated on 17 cases, including cases of bronchiectasis, traumatic pulmonary hæmorrhage, aneurysm, cancer, and sarcoma, both of the pleura and the lung, as well as tumours of the thoracic wall. Four cases were operated upon under increased pressure. The author finds that as the result of operating under such conditions exploratory thoracotomy is no more dangerous nor difficult than exploratory laparotomy. The use of such a chamber abolishes all risks of sudden dyspnœa, collapse, and the other unforeseen dangers which so frequently accompany extensive intra-thoracic operations. Even with an opening in the thorax of 20 centimetres and more, as is necessary in operations on the œsophagus, no change in the patient's condition can be observed. The author used Sauerbruch's and Brauer's apparatus of diminished and increased pressure respectively. He recommends that ether be used as the anæsthetic which he finds in no way dangerous. The patient should from time to time be allowed to respire a little oxygen given under pressure.

BABONNEIX and Bernard contribute an interesting study of the Ocular Manifestations of Chorea to the *Gazette des Hôpitaux*. Iritis has only once been recorded. Conjunctival anæsthesia is, however, fairly common; but, as Babinzki has shown, this must be attributed to involuntary suggestion on the part of the medical attendant. Pupillary symptoms are familiar to modern observers. Cadet has since recorded a striking case of choreiform movements of the iris, and Cruchet has called attention to the alternations of mydriasis and myosis often seen in chorea. The present authors, however, regard this symptom as inconstant and of no importance in the differential diagnosis between chorea and the various tics. Optic neuritis has been noted on several occasions, more especially by Bouchut, who attributes it to hyperæmia; but the suggestion has been advanced that the associated optic neuritis and choreic symptoms are both due to a meningitis. It has often been stated that the paralyses sometimes seen in chorea invariably spare the ocular muscles, and the authors cannot find a single instance of ocular paralysis in the literature of this subject.

VAN STOCKUM, in the *Zentralblatt für Chirurgie*, publishes two cases of Suprapubic Prostatectomy successfully performed without opening the bladder. His method is as follows: After making an incision above the pubes and cutting down on to the anterior wall of the bladder, this latter is carefully separated from the posterior surface of the pubic symphysis. This having been done, the retropubic tissues are stripped off the anterior surface of the prostate, and each lobe of the organ enucleated separately, after opening up the capsule by a vertical incision a little to one side of the middle line. Whilst enucleation is proceeding, an assistant, by means of a finger in the rectum, pushes the organ well forward. A catheter is next passed into the bladder and tied into position. A gauze plug is then put into the prostatic cavity, and a drainage tube inserted into the suprapubic wound. The author is of opinion, however, that the drainage tube is really unnecessary, and proposes for the future to sew up the wound, leaving only a small opening through which the end of the gauze plug is brought to the surface. The catheter is left in for about a fortnight. The author claims that this operation causes less damage to the tissues than that of Freyer, and reduces hæmorrhage to a minimum.

GILBERT BALLET, in a paper read before L'Académie de Médecine, is of opinion that the belief that the descendants of general paralytics are afflicted with a psychopathic or neurapathic taint is erroneous. An analysis of fifty cases of children of general paralytics, taken indiscriminately at ages from fifteen to thirty, only shows seven cases in which there was nervous or psychical derangement. Of these seven, two were epileptics, two were attacked with a psychosis of a doubtful nature, and three were extremely impressionable and emotional. From this, the author is led to believe that general paralysis is a disease of the nervous system only in so far as it has its seat therein and not by nature. Among the children of general paralytics nervous affections are the more common, the more frequently the children are born at a period long antecedent to the onset of the disease in the parent, which is to say, at a period nearer the initial syphilis of the parent. The nervous taint in such children is thus the direct outcome of hereditary syphilis, a fact which is well shown in certain cases of epilepsy, which seem to result from a specific meningitis in early childhood.

RECENT work by Roger suggests that, contrary to the general view, peptones do not represent the ultimate stage in the digestion of albumins. The substances absorbed by the organism, according to him, are not peptones, but bodies which fail to give the biuret reaction. He has experimented with finely minced muscle, added to water containing sulphuric acid, and kept at a temperature of 120° C. for 20 hours. With a 1-per-cent. solution of acid a part of the albumin is converted into peptone, and a much greater proportion of the albumin is transformed by a 2-per-cent. solution. If a 15-per-cent. solution be used, only abiuretic substances remain. It is interesting to note that the toxicity of these solutions varies directly as their content in peptones, which can be shown by injection into animals of the solutions after freeing them from acid by baryta and then filtering them. Peptones have the power of diminishing the blood-pressure, and it is interesting to note that in proportion as the solutions contain less peptone, so is their effect on the blood-pressure lessened, and a solution containing abiuretic substances only has no effect at all. Thus is explained the paradox of the toxicity of the absorbed products. Peptones which are toxic are not absorbed as such, but are turned into abiuretic bodies which are not toxic. Moreover, this fact explains also an observation formerly made by the same author and Garnier, who showed that the material contained in the duodenum is much more toxic than that contained in the lower part of the ileum, a fact which is explained by the gradual disappearance of the peptones as the chyme travels down the small intestine from the duodenum.

